# Stromal Cells Present in the Melanoma Niche Affect Tumor Invasiveness and Its Resistance to Therapy

**DOI:** 10.3390/ijms22020529

**Published:** 2021-01-07

**Authors:** Justyna Mazurkiewicz, Aleksandra Simiczyjew, Ewelina Dratkiewicz, Marcin Ziętek, Rafał Matkowski, Dorota Nowak

**Affiliations:** 1 Department of Cell Pathology, Faculty of Biotechnology, University of Wroclaw, Joliot-Curie 14a, 50-383 Wroclaw, Poland; aleksandra.simiczyjew@uwr.edu.pl (A.S.); ewelina.dratkiewicz@uwr.edu.pl (E.D.); dorota.nowak@uwr.edu.pl (D.N.); 2 Department of Oncology and Division of Surgical Oncology, Wroclaw Medical University, Plac Hirszfelda 12, 53-413 Wroclaw, Poland; zietek.m@dco.com.pl (M.Z.); rafal.matkowski@umed.wroc.pl (R.M.); 3 Wroclaw Comprehensive Cancer Center, Plac Hirszfelda 12, 53-413 Wroclaw, Poland

**Keywords:** tumor microenvironment, melanoma, invasion, drug resistance, cancer-associated fibroblasts, adipocytes, keratynocytes

## Abstract

Malignant melanoma is a highly metastatic type of cancer, which arises frequently from transformed pigment cells and melanocytes as a result of long-term UV radiation exposure. In recent years, the incidence of newly diagnosed melanoma patients reached 5% of all cancer cases. Despite the development of novel targeted therapies directed against melanoma-specific markers, patients’ response to treatment is often weak or short-term due to a rapid acquisition of drug resistance. Among the factors affecting therapy effectiveness, elements of the tumor microenvironment play a major role. Melanoma niche encompasses adjacent cells, such as keratinocytes, cancer-associated fibroblasts (CAFs), adipocytes, and immune cells, as well as components of the extracellular matrix and tumor-specific physicochemical properties. In this review, we summarize the current knowledge concerning the influence of cancer-associated cells (keratinocytes, CAFs, adipocytes) on the process of melanomagenesis, tumor progression, invasiveness, and the emergence of drug resistance in melanoma. We also address how melanoma can alter the differentiation and activation status of cells present in the tumor microenvironment. Understanding these complex interactions between malignant and cancer-associated cells could improve the development of effective antitumor therapeutic strategies.

## 1. Introduction

Melanoma is a tumor that arises from pigment cells, i.e., melanocytes, and is characterized by a high mortality rate among skin cancer patients. One of the major risk factors for melanoma is long-term exposure to UV radiation. In healthy skin, melanocytes cooperate with keratinocytes and other cells present in their vicinity to protect DNA from UV-induced damage, mainly in a melanin-dependent way [1]. Among the genetic aberrations that can arise following UV-irradiation, ca. 70% of mutations are present in genes encoding proteins associated with the mitogen-activated protein kinase (MAPK) pathway, e.g., *BRAF*, *NRAS* [2]. About 50% of melanoma patients exhibit a mutation in the *BRAF* gene (*BRAF* V600E), which leads to the appearance of a constitutively active kinase [3,4]. Currently, there are multiple clinically approved therapies targeting melanoma-specific molecular markers, including mutated *BRAF*. However, in patients subjected to such treatments, resistance to therapy develops rapidly [5,6].

**Figure 1:**
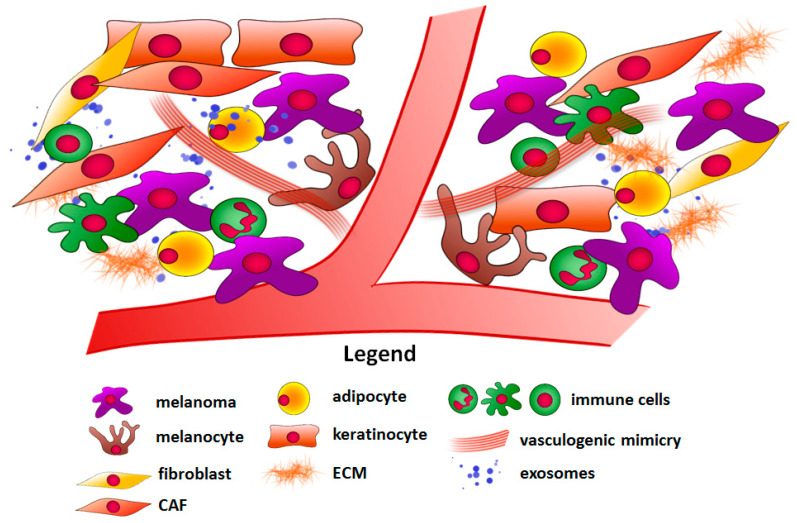
Components of the melanoma microenvironment. Abbreviations: CAF, cancer-associated fibroblast; ECM, extracellular matrix.

One of the factors that influence anticancer therapy effectiveness is the tumor microenvironment. It is important to note that solid tumors consist not only of malignant cells but also of adjacent cells such as cancer-associated fibroblasts (CAFs), keratinocytes, adipocytes, and immune cells (Figure 1). Other elements such as physical factors (hypoxia) and components of the extracellular matrix (ECM) present in the tumor niche should also be taken into consideration [7,8]. The cancer microenvironment has a diverse impact on melanoma development and resistance to therapy. Firstly, it can act as a physical barrier, which reduces drug delivery to cancer cells [9]. Secondly, melanoma neighboring cells can facilitate tumor growth and enhance its angiogenesis and invasive abilities in a paracrine way. They produce growth factors, cytokines, chemokines, etc. that affect other cells in their vicinity, and even in distant parts of the body [10]. Additionally, they secrete matrix metalloproteinases (MMPs), which mediate the ECM degradation, thus allowing cancer cells to invade through the tissue [11]. Cells present in the tumor niche can also produce high-energy compounds, which then can be used by melanoma [12]. Finally, immune cells, which in normal conditions help the organism to combat malignantly transformed cells, are able to support the immune escape of melanoma cells employing a variety of mechanisms involving expression of inhibitory receptors, secretion of pro-inflammatory factors, or induction of pro-tumoral immune cell phenotype [13].

The above-described aspects of the tumor niche contribute to the melanoma aggressiveness and reduced response to the treatment, suggesting that cancer-associated cells could emerge as new therapeutic targets.

In this review, we would like to focus on the influence of cells present in the melanoma niche on cancer progression and the appearance of therapy resistance mechanisms. Here, we will give special attention to keratinocytes, cancer-associated fibroblasts, and adipocytes, as we have already described the role of immune cells in melanoma development in our previous review [13].

## 2. Keratinocytes

**Figure 2:**
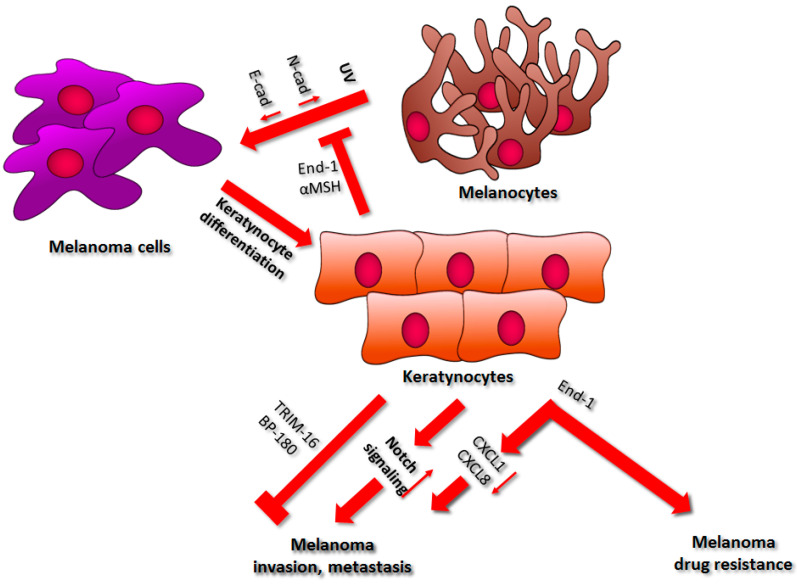
The influence of keratinocytes on melanoma development. A detailed description of the processes regulated by keratinocytes can be found in the main text. Abbreviations: UV, ultraviolet; N-cad, N-cadherin; E-cad, E-cadherin; End-1, endothelin-1; CXCL1, C-X-C-motif chemokine 1; CXCL8, C-X-C-motif chemokine 8; TRIM16, Tripartite motif-containing protein 16.

In the human epidermis, the proliferation and localization of melanocytes are regulated by their interplay with keratinocytes. It is estimated that a single melanocyte is connected to ca. thirty-six keratinocytes. They protect pigment cells from transformation into melanoma cells through direct interaction and in a paracrine manner via secretion of endothelin-1 (End-1) and α-melanocyte-stimulating hormone (α-melanocortin, αMSH) (Figure 2) [14,15]. In melanocytes, these two molecules bind to their specific receptors: endothelin B receptor and melanocortin-1 receptor, respectively. This, in turn, leads to the activation of DNA damage sensors and proteins involved in the process of DNA damage repair (e.g., activating transcription factor 2 (ATF2) and p53), which eventually accelerates global genome repair [16]. Kadekaro et al. showed that End-1 and αMSH regulate melanocyte survival through activation of the inositol triphosphate kinase-AKT pathway, as well as via the inhibition of the UV-induced reduction in Bcl-2 (B-cell lymphoma 2) expression. They also enhance MITF (microphthalmia-associated transcription factor) production, thus promoting melanocyte proliferation [17]. These facts confirm the preventive role of keratinocyte-derived End-1 and αMSH against UV-induced melanomagenesis [16].

Along with UV radiation, some other factors could trigger melanomagenesis as a consequence of defective keratinocyte control of melanocytes. Gonzalez et al. have shown that under continual arsenic exposure, keratinocytes express elevated levels of several small non-coding RNAs, called microRNAs (miRs), namely miR-21, miR-200a, and miR-141, which are involved in the regulation of signaling pathways promoting melanoma growth [18]. Upregulation of miR-21 following UV irradiation was detected in keratinocytes, epidermal cells, fibroblasts, melanoma cells, and keratinocyte-derived exosomes and was correlated with gene expression associated with cell survival and sustained proliferation (e.g., phosphatidylinositol 3 kinase (PI3K) and p53), angiogenesis (e.g., hypoxia-inducible factor 1-α (HIF1α)) and cancer-cell-invasive abilities (e.g., tissue inhibitor of metalloproteinanses-3 (TIMP3)) [19]. Expression of miR-200a and miR-141 was downregulated upon αMSH stimulation in B16-4A5 mouse melanocytes, while overexpression of these non-coding RNAs led to reduced melanogenesis and directly influenced MITF level [20]. Lastly, phosphatase and tensin homolog (PTEN), a negative regulator of the PI3K/AKT pathway, is one of the targets of miR-141, which was also reported to be upregulated in keratinocytes following UV exposure [21].

On the other hand, it was reported that melanoma is also able to influence keratinocyte differentiation. Treatment of keratinocytes with conditioning medium derived from melanoma cells led to a decrease in the expression of a differentiating marker—keratin 10, specific for suprabasal cells undergoing cornification and subsequent shedding, and elevated keratin 14 production, which is characteristic for intensively proliferating basal keratinocytes [22].

### 2.1. Regulation of Cell–Cell Interactions

Keratinocytes control pigment cell functions via secreted growth factors and through adhesion molecules, including cadherins, which are transmembrane proteins that play a role in cell–cell adhesion [14,23,24,25]. E-cadherin cytoplasmic domain is connected to the cell cytoskeleton through the protein complex containing β-catenin [26]. The extracellular domain interacts with similar cadherins located on the surface of other cells. Downregulation of E-cadherin and upregulation of N-cadherin let melanocytes escape out of keratinocyte control (Figure 2). Pigment cells are then able to interact with other cells that express N-cadherin, e.g., endothelial cells and fibroblasts [27].

Changes in the respective cadherin transcription level are driven by epithelial–mesenchymal transition (EMT) regulators (e.g., Snail, Slug, Twist), and such a cadherin switch could induce melanoma tumorigenesis [23]. Hepatocyte growth factor (HGF), which is produced by several types of stromal cells, was shown to stimulate the expression shift between E- and N-cadherin. However, the final result depends on the cancer stage [28]. It was demonstrated that N-cadherin upregulation facilitates melanoma invasion, as it enables cancer cells to connect with vascular endothelial cells, whose interplay is necessary for intra- and extravasation processes [29,30,31]. N-cadherin also supports the survival of melanocytes through the suppression of proapoptotic factor production mediated by AKT pathway activation, which then enhances the β-catenin level and, thus, inactivates Bad (Bcl-2-associated agonist of cell death)—the proapoptotic molecule [25,30]. Moreover, the renewal of E-cadherin expression in melanoma cells leads to their reconnection with keratinocytes, inhibits invasion, and induces apoptosis [32]. It was reported that isolated melanocytes in mono-culture conditions acquired new features such as expression of melanoma-associated proteins (e.g., MUC18, β3 integrin subunit), bi- or tripolar morphology, or enhanced proliferation [32,33]. However, the co-culture of pigment cells with basal keratinocytes, but not differentiated cells, restored normal melanocyte properties. Additionally, it was shown that melanoma cells are resistant to this process [32,34].

Hsu et al. have demonstrated that forced E-cadherin expression in melanoma cells led to the formation of cancer cell–keratinocyte interactions. E-cadherin-expressing melanoma cells also exhibited decreased cell growth and colony formation rate. When co-cultured with keratinocytes, the level of melanoma-specific proteins such as invasion-related MUC18 or β3 integrin subunit in these cells was undetectable compared to monoculture. Using a three-dimensional in vitro skin model composed of a dermal compartment (a layer of fibroblasts in a collagen gel), an epidermal layer (melanoma cells with keratinocytes), and a basement membrane, Hsu et al. reported changes in cancer cell localization. Control melanoma cells were located in the deep dermal layer, whereas E-cadherin-expressing cells stayed in the epidermis or the upper part of the dermis and showed properties of apoptotic cells [32].

It was demonstrated that melanoma cells retain the expression of the endothelin B receptor, thus allowing for End-1-mediated stimulation [35]. Endothelin-1 was shown to downregulate E-cadherin expression in melanoma through activation of caspase-8, thus contributing to cancer invasion (Figure 2) [36]. It also induced the secretion of metastasis-inducing CXCL1 (C-X-C- motif chemokine 1) and CXCL8 (C-X-C- motif chemokine 8) in melanoma cells, while in vivo studies indicated a correlation between End-1 level and melanoma invasiveness [37,38,39,40]. Therefore, anticancer therapies using antibodies or nanobodies targeting the endothelin-1 receptor are considered a viable treatment strategy against melanoma [41,42].

### 2.2. Drug Resistance

Melanoma tumors exhibit a high rate of heterogeneity, as they consist of cells characterized by either a more proliferative (MITF^HIGH^/AXL^LOW^) or a more invasive (AXL^HIGH^/MITF^LOW^) phenotype [43]. There is a positive correlation between the occurrence of AXL^HIGH^ cells and resistance to inhibitors targeting the MAPK pathway in patients carrying a *BRAF* or *MEK* (mitogen-activated protein kinase kinase) mutation [44]. It was demonstrated that keratinocyte-derived endothelin-1 is required for AXL-induced resistance and targeting the endothelin B receptor led to increased sensitivity in BRAF inhibitor-resistant cells (Figure 2) [16,45].

### 2.3. Factors Secreted by Keratinocytes

Keratinocytes are also able to influence melanocytes and melanoma cells through factors secreted to the stroma. Under the influence of UV radiation, keratinocytes secrete tripartite motif-containing protein 16 (TRIM16) [46]. It was reported that the TRIM16 level in melanoma was lower compared to the normal melanocytes, which also correlated with a rate of lymph node metastasis in TRIM16^LOW^ melanoma patients. Moreover, BRAF inhibitor treatment increased the TRIM16 production in melanoma cells, while TRIM16-deficient mice exhibited elevated incidence of metastasis compared to the control animals [46,47]. This study supports the thesis that keratinocyte-derived TRIM16 may inhibit melanoma metastasis (Figure 2) [46].

Moreover, basal keratinocytes express BP180/collagen XVII, which is a cell-matrix adhesion protein related to different types of skin cancers, including melanoma [48,49]. Hwang et al. showed that keratinocytes derived from BP180-deficient mice exhibited the upregulation of CXCL1 expression compared to control animals, in which cytokine acts as a chemoattractant for myeloid-derived suppressor cells (MDSCs). It was reported that inhibition of MDSC influx in BP180-deficient mice resulted in a reduction of tumor volume and the metastasis rate of B16 melanoma cells. These results validate the antitumor role of BP180 in melanoma (Figure 2) [48].

Keratinocytes exposed to UV radiation not only seem to display anticancer properties but are also able to support melanoma progression. During melanoma development, two different growth phases are distinguished: the radial growth phase (RGP), in which melanoma cells proliferate slowly, and the vertical growth phase (VGP), which is characterized by a fast proliferation rate and the formation of metastases [50]. One of the theories concerning the change of melanoma progression stage involves the interaction of cancer cells with distant differentiated keratinocytes, which express Notch ligands. These molecules then bind to the receptors present on melanoma cells and activate Notch signaling, leading to the abolition of the MITF-mediated inhibition of miR-222/221 expression and subsequent increase in melanoma invasion [51]. Moreover, Li et al. have demonstrated that the serum level of miR-221 has a prognostic value in patients suffering from cutaneous melanoma [52]. It was also shown that the upregulation of miR-222 in melanoma leads to the activation of the PI3K/AKT signaling pathway and the reduction of expression of p27^Kip1^ protein, which is the cell cycle inhibitor [52]. Similarly, in uveal melanoma, this miRNA led to elevated cell proliferation and migration in a PI3K/AKT MMP9 (matrix metalloproteinase 9)-dependent manner [53]. Furthermore, UV-induced keratinocytes secrete tumor necrosis factor alpha (TNFα) and interleukin 1β (IL-1β) [54]. These factors then induce the secretion and activation of MMP9 in VGP melanoma. However, MMP9 is detected only in VGP melanoma, not in RGP tumors. Hence, this enzyme is considered a VGP marker. Based on these results, it was postulated that TNFα and IL-1β secreted by keratinocytes could be involved in an RGP/VGP melanoma phase switch [55].

## 3. Cancer-Associated Fibroblasts

**Figure 3:**
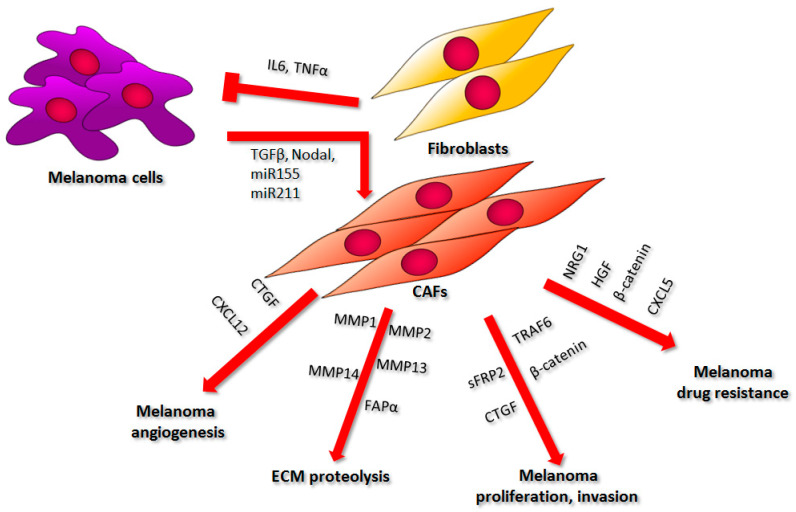
The influence of fibroblasts on melanoma cell proliferation, invasion, ECM remodeling, angiogenesis, and drug resistance. A description of the presented phenomena can be found in the text. Abbreviations: IL6, interleukin 6; TNFα, tumor necrosis factor α; TGFβ, transforming growth factor β; miR155, microRNA 155; miR211, microRNA 211; CTGF, connective tissue growth factor; CXCL12, C-X-C-motif chemokine 12; MMP1, matrix metalloproteinase 1; MMP2, matrix metalloproteinase 2; MMP13, matrix metalloproteinase 13; MMP14, matrix metalloproteinase 14; FAPα, fibroblasts activating protein α; sFRP2, freezled-related protein 2; TRAF6, TNF receptor-associated factor 6; NRG1, neuregulin1; HGF, hepatocyte growth factor; CXCL5, C-X-C-motif chemokine; ECM, extracellular matrix; CAFs, cancer-associated fibroblasts.

Fibroblasts are a major component of the melanoma niche and may constitute up to 80% of the tumor mass [56]. Normal fibroblasts can inhibit cancer growth and development at tumor onset. Through the secretion of factors such as interleukin 6 (IL-6) or interferon gamma (IFNγ), which are responsible for immune cell mobilization, they can indirectly suppress tumor progression (Figure 3) [57]. Moreover, fibroblasts regulate the dynamics of ECM content. They supply matrix structural components such as types I, III, and IV collagen, fibronectin, elastin, tenascin C, and proteoglycans, as well as enzymes that catalyze ECM element degradation like metalloproteinases [58]. Melanoma cells require the latter proteolytic activity to form metastases, while inhibition of the ECM degradation by normal fibroblasts leads to the reduction of melanoma’s invasive abilities [58,59].

Melanoma cells can switch the phenotype of the cells present within the tumor niche toward the more supportive one for cancer survival. It was shown that melanoma can change the metabolism of neighboring fibroblasts, leading to an increased anaerobic glycolysis rate, which results in the production of high-energy compounds later used by cancer cells. This effect can be achieved, among other ways, through the regulation of metabolic pathways by small microRNAs released by melanoma cells in exosomes, particularly miR-155 and miR-210 [12]. However, the role of melanoma-derived miR-155 in fibroblast reprogramming is even more extensive. It can also elicit proangiogenic activity through the upregulation of vascular endothelial growth factor a (VEGFa), fibroblasts growth factor 2 (FGF2), and MMP9 expression, thus facilitating tumor progression (Figure 3) [60]. Additionally, transforming growth factor β (TGFβ), which is also secreted by melanoma cells, can convert normal fibroblasts into cancer-associated fibroblasts (Figure 3) [61]. These fibroblasts, present in the tumor vicinity, are large spindle-shaped cells with similar characteristics to myofibroblasts, which are observed in areas of chronic inflammation and wound healing. Both cell types can be identified by the elevated level of alpha smooth muscle actin (αSMA); however, not all CAFs express this protein. Some populations of CAFs can be recognized based on the expression of other markers, e.g., fibroblast activation protein alpha (FAPα) or fibroblast-specific protein 1 (FSP1) [62,63,64,65]. Another molecule, which is thought to transform normal fibroblasts into cancer-associated ones, is Nodal—a member of the TGF superfamily. Expression of this marker is correlated with an increased αSMA level in melanoma tumors, while the treatment of normal fibroblasts with Nodal leads to their differentiation into CAF (Figure 2) [66]. Kuninty et al. also indicated the existence of a link between CAFs markers (e.g., αSMA, collagen, platelet-derived growth factor receptor β (PDGFβR)) and miR expression. They showed that the inhibition of miR-199a/-214 in the pancreatic precursor of CAFs inhibited the expression of TGFβ-induced differentiation markers [67]. These particular miRs, along with miR-155, were also found in melanoma-derived extracellular vesicles and could potentially influence fibroblasts present in the melanoma niche [68].

Interestingly, melanoma can drive the fibroblast phenotype switch through released miRs not only in adjacent cells but also in distant ones [69]. It was shown that fibroblast reprogramming toward CAFs may be facilitated by melanoma-derived miR-211 delivered to these cells not in exosomes, but in melanosomes, in which melanin is produced and then transported to keratinocytes (Figure 3). This non-coding RNA affected insulin-like growth factor 2 receptor (*IGF2R*) expression and, thus, led to the MAPK signaling pathway upregulation, which resulted in elevated proliferation, migration, and increased production of proinflammatory proteins (IL-1β, Il-6, IL-8, CXCL1, CXCL2, and COX2 (cyclooxygenase 2)) [70]. Interestingly, Dror et al. reported that melanosomes and exosomes share 70% of miRNAs, while in another study, vemurafenib treatment of melanoma cells and xenografts caused the upregulation in miR-211 secretion encapsulated in extracellular vesicles released by examined cells [70,71].

Aside from normal fibroblasts, CAFs can arise from pre-adipocytes, myofibroblasts, bone marrow-derived progenitor cells, smooth muscle cells, and they can support tumor development in various ways [62].

### 3.1. Cell Proliferation, Invasion, and Metastasis

CAFs play an important role in the proliferation and invasion of melanoma cells. One of the mediators of their influence on tumor growth and progression is β-catenin, a potential target for melanoma treatment by its participation in cell adhesion and regulation of gene expression through Wnt signaling [58]. It was indicated that melanoma tumors consisting of cancer cells and β-catenin-deficient fibroblasts grew faster and reached a larger volume as compared to the control ones (Figure 3). This observation confirms the anticancer effect of normal fibroblasts at the onset of melanoma development. Nevertheless, in the case of already existing melanoma tumors, knockout of β-catenin in CAFs has an opposite effect and results in the reduction of cancer growth [72]. In tumors containing β-catenin-deficient CAFs, the level of CAF-specific markers (e.g., αSMA) was decreased compared to the control, which correlated with a reduced responsiveness of fibroblasts to melanoma-derived activating signals. Moreover, CAFs devoid of β-catenin exhibited reduced ERK (extracellular signal-regulated kinase)/MAPK and PI3K/AKT signaling, as well as blocked EMT in BRAF-mutated melanoma cells. They also induced G1/S cell cycle arrest in cancer cells through the downregulation of cyclin (A2, B1, D1, D3, E) levels and the upregulation of cyclin-dependent kinase inhibitor (p16, p18, p27, p57) expression [73]. In addition, sFRP2 (freezled-related protein 2), a β-catenin inhibitor secreted predominantly by aged fibroblasts compared to young ones, increased melanoma cell invasion in an indirect co-culture system [74].

Activated fibroblasts also influence melanoma metastasis. CAFs express connective tissue growth factor (CTGF), which belongs to the CCN (CYR62, CTGF, NOV) family [75,76]. CTGF expression is induced by cytokines, mitogens, hormones, oxygen loss, and growth factors. The main molecule stimulating CTGF production is TGFβ, which is also involved in processes such as fibroblast proliferation, wound healing, fibrosis, and the regulation of ECM composition [77]. Moreover, CTGF promotes integrin-based adhesion, thus regulating the interactions between cells present in the tumor niche. Connective tissue growth factor can also bind to cytokines and modulate their activity, thereby acting as a mediator initiating signaling pathways induced by these molecules [78,79]. It was demonstrated that CTGF is overexpressed in malignant melanoma, as well as in activated fibroblasts present within the tumor niche, while the loss of CTGF expression led to a decrease in fibroblast activation level [80,81]. Additionally, Hutchenreuther et al., using a syngenic mouse model, have shown that depletion of CTGF in the tumor stroma disturbs melanoma cell metastasis in vivo (Figure 3) [81].

Another protein important to melanoma-associated fibroblast activity is TNF receptor-associated factor 6 (TRAF6). TRAF6 shows an E3 ubiquitin ligase activity and is expressed at a high level in melanoma [82,83,84]. It interacts with extracellular matrix metalloproteinase inducer CD147(cluster of differentiation 147)/Basigin (BSG), changing its membrane localization and, thus, leading to the upregulation of MMP expression. Therefore, TRAF6 can enhance melanoma invasion and metastasis through the regulation of MMP9 production [84]. Furthermore, TRAF6 is needed for the activation of AKT kinase, which is involved in cancer progression [85,86,87]. It catalyzes AKT ubiquitination, hence promoting its membrane localization, which is crucial for the phosphorylation and subsequent activation of this kinase [85]. Moreover, TRAF6 is upregulated in αSMA-expressing fibroblasts. It was shown that conditioned medium collected from fibroblasts overexpressing TRAF6 increased melanoma proliferation, migration, and invasion (Figure 3). TRAF6-deficient fibroblast-derived conditioned medium had an opposite effect on melanoma, as it led to a decrease in cancer cell migration, invasion, and cell growth. Similar results were obtained in vivo [88].

Moreover, metalloproteinases such as MMP1, MMP2, MMP13, and MMP14 secreted by CAFs facilitate melanoma invasion by participation in ECM digestion and, thus, the formation of a pathway used by cancer cells to move through the tissues (Figure 3) [59,89,90,91,92,93]. ECM remodeling is also promoted by FAP, a member of the serine protease family, overexpressed by activated fibroblasts (Figure 3) [94,95]. FAP is also thought to suppress T cell recruitment and function and, therefore, support melanoma immune escape [96,97]. Moreover, it was confirmed that the loss of β-catenin expression in CAFs correlates with a decrease in fibronectin and collagen levels in the tumor stroma. These proteins are components of the tumor scaffold; thus, changes in their levels correspond with reduced cancer growth [73].

### 3.2. Angiogenesis

Cancer-associated fibroblasts also contribute to melanoma angiogenesis, which is a process crucial for the intensively growing tumor mass with a high demand for oxygen and nutrients. A CAF-derived cytokine, CXCL12, interacts with CXCR4 (C-X-C-motif chemokine receptor 4), a protein overexpressed on the surface of cancer cells, whose interplay leads to the induction of the tumor angiogenesis through endothelial cell recruitment into the tumor niche (Figure 3). Interaction between CXCL12 and CXCR4 also facilitates the adhesion of cancer cells to microvascular endothelial cells via an integrin β_1_-based mechanism, which eventually results in an elevated rate of melanoma pulmonary metastases [59,98,99,100,101].

An important factor, which is produced by CAFs and has an influence on melanoma angiogenesis, was mentioned previously as one of the inductors of melanoma invasiveness (Figure 3). It was shown in vivo that CTGF-knockout in mouse fibroblasts resulted in reduced neovascularization induced by the tumor, which was additionally confirmed by a decreased level of endothelial marker CD31/platelet endothelial cell adhesion molecule (PECAM-1) within the tumor and its stroma. Moreover, mice with CTGF-ablated fibroblasts and injected with B16-F10 melanoma cells displayed impaired vasculogenic mimicry—a phenomenon characterized by the rearrangement of cancer cells into vessel-like structures typical reported in the case of endothelial cells [75].

### 3.3. Effect of CAFs on Melanoma Drug Resistance

Activated fibroblasts affect the acquisition of drug resistance in melanoma in various ways, a.o., through the secretion of growth factors, including HGF, insulin-like growth factor 1 (IGF1), and basic fibroblast growth factor (bFGF), which support cancer cell growth and proliferation [59,102]. HGF is also secreted by CAFs upon BRAF kinase inhibitors’ (BRAFi) treatment and, thus, contributes to the therapy resistance (Figure 3). It has been reported that patients suffering from melanoma with detected HGF secretion by tumor stroma exhibited a much weaker response to BRAFi treatment compared to patients negative for stroma-derived HGF. It remains unclear as to whether this effect is caused by the upregulation of HGF in fibroblasts following BRAFi therapy or whether the use of these inhibitors results in the recruitment of HGF-expressing fibroblasts into the melanoma niche [103].

β-catenin, which plays a role in melanoma proliferation and invasiveness, and has already been described in one of the previous sections, also influences tumor drug resistance (Figure 3). It was demonstrated that following the co-culture of melanoma cells with aged fibroblasts, the expression of MITF and a redox effector, APE1 (apurinic/apurymidinic endonuclease 1), was decreased in a β-catenin-dependent way. The reduction of APE1 in melanoma cells resulted in a weaker DNA damage response to reactive oxygen species (ROS) [74]. Elevated ROS level, as well as a decreased β-catenin and MITF expression, is connected to BRAFi resistance [104,105,106,107,108]. However, ROS inhibition with acetylcysteine in aged fibroblasts led to melanoma cell death in co-culture conditions. This suggests that BRAFi-resistant cells may be extremely sensitive to antioxidants, while they are under the influence of the aged microenvironment. Moreover, in clinical studies, patients who responded better to BRAFi treatment exhibited a higher level of β-catenin compared to those with a weaker response [74].

It has been indicated that epidermal growth factor receptors (ERBB) participate in the acquisition of resistance to BRAFi, and the level of ERBB3 protein is increased in resistant melanoma cells [109]. CAF-derived neuregulin 1 (NRG1) is an ERBB3 ligand [110,111]. The NRG1 level in melanoma cells was very low or undetectable, whereas in normal and cancer-associated fibroblasts it was relatively high. Furthermore, the fibroblast-derived conditioned medium (CM) activated ERBB3-dependent pathways in cancer cells incubated with vemurafenib. Under the influence of CM derived from NRG1-depleted fibroblasts, melanoma cells exhibited an increase in ERBB3 signaling at a much lower level compared to the control. These data suggest that NRG1 secreted by fibroblasts enhances the growth of BRAFi-resistant melanoma cells (Figure 3) [112]. Moreover, in vivo studies showed that the combination of ERBB3 and MEK inhibitors led to a more significant reduction in tumor growth compared to treatment with MEKi as a monotherapy [112,113].

CXCL5 is a cytokine that also participates in melanoma drug resistance acquisition (Figure 3). A correlation between the expression of αSMA and programmed cell death ligand-1 (PD-L1) in human tumors was recently reported, which suggests that CAFs could influence the expression of PD-L1 in cancer cells and, thus, facilitate melanoma immune escape [114,115]. Li et al. confirmed this hypothesis utilizing the co-culture of CAFs isolated from mice tumors and of B16 melanoma cells. CAFs exhibited increased PD-L1 expression and produced CXCL5 cytokine at a much higher level than normal fibroblasts [114]. Under the influence of CXCL5-overexpressing CAFs, melanoma cells displayed an elevated level of PI3K/AKT activation and PD-L1 expression in a CXCR2-dependent way [114].

## 4. Adipocytes

Another important element of the tumor microenvironment that affects the progression of melanoma is adipose tissue, which constitutes the main component of the deepest layer of the skin, the hypodermis [116]. It is composed mainly of adipocytes, but also contains a stromal vascular fraction including endothelial cells, macrophages, pericytes, monocytes, and pluripotent stem cells [117,118].

**Figure 4:**
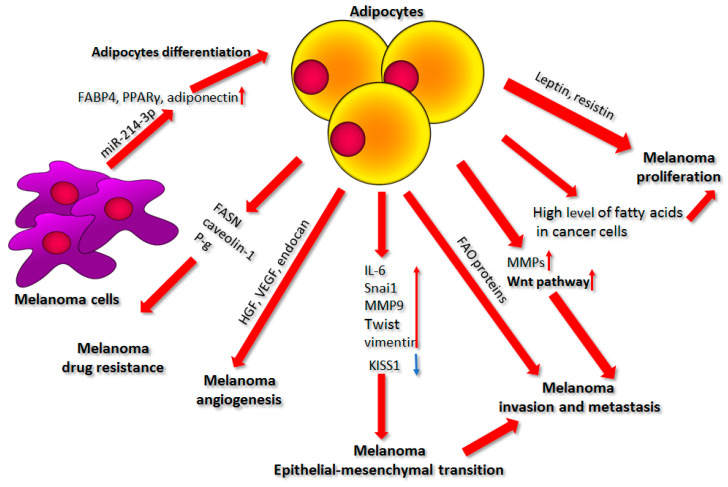
The influence of adipocytes on melanoma proliferation, epithelial–mesenchymal transition, cell invasion, metastasis, angiogenesis, and drug resistance. A detailed description of the shown processes is present in the text. Abbreviations: FABP4, fatty acid binding protein 4; PPARy, peroxisome proliferator-activated receptor γ; miR-214-3p, microRNA 214-3p; FASN, fatty acid synthase; P-g, P-glycoprotein; HGF, hepatocyte growth factor; VEGF, vascular endothelial growth factor; IL-6, interleukin 6; MMP9, matrix metalloproteinases 9; FAO, fatty acid oxidation; MMPs, matrix metalloproteinases.

Differentiated white adipocytes specialize in the accumulation of lipids, primarily in the form of triacylglycerol, which can be later released in the form of free fatty acids (FFA). Until recently, adipose tissue was primarily assigned the role of lipid storage. Current knowledge allows us to recognize it also as an inflammatory and hormonal organ [119,120]. Adipocytes secrete factors collectively termed adipokines, which include inflammatory factors (IL-6, IL-11, leukemia inhibitory factor (LIF), and plasminogen activator inhibitor-1 (PAI-1)), metabolic markers (insulin-like growth factor-binding protein (IGFBP), FGF-21), angiogenic growth factors (endocan, HGF, VEGF, IGF-I), and hormones (leptin, resistin, retinol-binding protein 4 (RBP-4)) (Figure 4) [121]. Cancer cells express receptors capable of binding a majority of these molecules [122]. The above-mentioned adipokines activate numerous signaling pathways including PI3K/AKT, MAPK, and JAK(Janus kinase)/STAT(signal transducer and activator of transcription), which eventually supports cancer cell growth, proliferation, invasion, and resistance to apoptosis by controlling the activation of proteins involved in tumor progression [123].

The presence of adipocytes, especially in those who are “obese”, leads to poorer prognosis for patients, less effective treatment results, and more cancer-related deaths [121]. In obese patients, the adipose tissue expands as a result of increased adipocyte size associated with elevated triglyceride storage and a raised amount of adipose progenitor proliferation and differentiation [116,124]. Obesity is accompanied by the ability of hypertrophied adipocytes to secrete elevated amounts of proinflammatory adipokines, including monocyte chemoattractant protein (MCP)-1, TNF-α, IL-6, IL-8, PAI-1, leptin, extracellular matrix remodeling proteins, and free fatty acids. These factors significantly alter the adipose tissue microenvironment and can influence cancer progression [117,125].

Recent reports show that there may exist a positive correlation between the increase in body fat and the risk of skin melanoma among patients [126,127]. It has also been demonstrated that obesity stimulates the growth of this tumor in mice [128,129]. Jung et al. showed that high-fat-diet-effected obesity stimulates cancer growth as well as metastases in the lung and lymph nodes in mice injected with melanoma cells [129,130]. Similar results were obtained by Pandey et al., who indicated that diet-induced obesity enhances melanoma progression, which is connected to the elevated level of caveolin 1 (Cav-1) and fatty acid synthase (FASN). FASN is a key enzyme in the de novo synthesis of fatty acids, which plays a crucial role in the proliferation and survival of cancer cells, as it provides cells with fatty acids for energy generation and ensures their proper membrane architecture. Cav-1 is a membrane-associated protein participating in signal transduction and maintenance of cell membrane shape. Cav-1, stabilized by FASN by palmitoylation, is involved in drug resistance and therefore constitutes a tumor-promoting factor in melanoma (Figure 4) [129,131,132].

### 4.1. Factors Secreted by Adipocytes

A key role in the stimulation of melanoma progression by adipocytes is played by factors secreted by the latter cells. Coelho et al. showed that components released by adipocytes or exposure to adipose tissue- CM increase the ability of melanoma cells to grow, multiply, migrate, spread, and avoid apoptosis [121]. Furthermore, they indicated that the patterns of secretion are different in the CMs collected from subcutaneous adipose tissue (SAT) and visceral adipose tissue (VAT). Visceral adipose tissue in relation to subcutaneous adipose tissue showed a higher expression of FGF21 and IGFBP-5 and decreased levels of HGF, IGF-I, IGFBP-2, IGFBP-3, and IGFBP-6 [121]. The authors also showed that SAT and VAT exert distinct effects on melanoma aggressiveness. SAT CM enhances melanoma cell motility and anchorage-independent proliferation, while VAT prominently improves the adhesion of these cells to the substrate [121].

Epidemiological studies demonstrated that a high level of leptin in serum is positively correlated with the risk of melanoma, while in vivo, injection of leptin into melanoma-bearing mice led to the increase in weight and size of the tumor [128,133]. It was shown that both leptin and resistin, two hormones released by adipocytes, stimulate melanoma cell proliferation in vitro by regulating FASN as well as the AKT-based signal transduction pathway (Figure 4) [123,134].

Another adipokine, adiponectin, plays an opposite role in melanoma, as its low level is associated with obesity. This molecule is downregulated in melanoma patients. Furthermore, it reduces cell growth and induces apoptosis in cancer cells, which is caused mostly by the activation of the AMPK (AMP-activated protein kinase) signaling pathway [116,135].

### 4.2. Source of Nutrients

It is well known that lipid metabolism is important for cancer nutrition because it is connected to the cancer cells’ requirement for energy and structural elements essential for rapid proliferation. Primarily, modifications in lipid metabolism were assigned to the genetic and epigenetic changes in cancer cells connected to their metabolic reprogramming [136,137].

Recently, Zhang et al. showed that adipocytes can directly transfer lipids to melanoma cells in vivo. Increased lipid content in melanoma is achieved via lipid uptake, rather than the increase in de novo lipogenesis. There is a well-known model in which cancer cells induce metabolic changes in adipocytes, resulting in enhanced lipolytic activity and the release of fatty acids, which then leads to cancer-associated cachexia in patients. Cachexia is a phenomenon common in cancer patients with advanced disease and stemming from adipose tissue atrophy, which is associated with increased lipolysis, disturbances in the proper storage of triacylglycerols, and free fatty acid and glycerol release [117,138].

Cancer cells growing in close proximity to adipocytes modify their metabolism to exploit metabolites that are both produced by these cells and present in the tumor microenvironment, to the maximum extent [117,137]. Zoico et al. confirmed that adipocytes serve as a source of nutrients for melanoma cells. They found that after a few days of cancer cell and 3T3-L1 adipocyte co-culture, the latter were reduced in number, size, and lipid droplet content, which was potentially connected to the lipid transfer to melanoma cells [139]. Moreover, Kwan et al. noted that melanoma cells co-cultured with adipocytes exhibited an elevated level of fatty acids, particularly palmitic acid, which stimulated cancer cell proliferation, influenced cell cycle distribution, and increased activation of AKT- and PI3K-based signaling pathways [140]. Adipocyte-derived lipids are transferred to melanoma cells through the FATP (fatty acid transport protein)/SLC27A (solute carrier family 27) family of lipid transporters. Zhang et al. established that melanoma cells overexpress one of these proteins—fatty acid transport protein 1 (FATP1), which contributes to the increased lipid uptake and tumor cell proliferation in vivo. Downregulation of FATP1 following the application of a small-molecule FATP inhibitor called Lipofermata resulted in a notable reduction in lipid content, as well as in melanoma cell growth and invasion [136,141].

### 4.3. Cell Invasion and Metastasis

The influence of adipocytes on melanoma motility was further investigated by Zoico et al. They noticed that as a result of the co-culture of melanoma cells and adipocytes, the latter were replaced by dedifferentiated cells with a fibroblast-like phenotype. This occurrence was also accompanied by the reduced expression of adipocyte-specific proteins (like adiponectin and glucose transporter type 4 (GLUT4)), and an elevated level of fibroblast-specific markers (like collagen, MMPs, and α-SMA) [139].

Moreover, melanoma cells co-cultured with adipocytes demonstrated increased migratory abilities associated with the activation of the Wnt pathway (also upregulated in obesity). Following the Wnt binding to its receptor, AKT kinase undergoes phosphorylation, which results in β-catenin translocation to the nucleus and activation of the expression of genes promoting the invasion of melanoma cells such as encoding LEF-1 (lymphoid enhancer binding factor 1)—a transcription factor stimulating the migration, angiogenesis, and metastasis of cancer cells [139]. Furthermore, it was indicated that the increased cell proliferation, migration, and invasion arising under the influence of adipocytes-conditioned medium was connected to the elevated activity of matrix metalloproteases MMP9 and MMP2 in melanoma cells [142]. These MMPs are also secreted by the adipocytes themselves, especially the “obese” ones [143]. Stimulation of the invasive abilities of melanoma cells by adipocytes was also associated with an elevated expression of numerous oncogenic proteins in cancer cells like cyclooxygenase 2, cyclin D1, and cell survival proteins (Bcl-2, Bcl-xL, Mcl-1 (myeloid cell leukemia 1), survivin, and IAP-2 (inhibitor of apoptosis protein-2), as well as with the activation of AKT/mTOR (mammalian target of rapamycin) signaling pathway (Figure 4) [142].

In addition, skin adipocytes promote metastasis by sensitizing melanoma cells to TGFβ. Golan et al. demonstrated that adipocytes co-cultured with melanoma secreted IL-6 and TNF-α, which induced a proliferative-to-invasive phenotypic switch in cancer cells by the repression of miR-211 expression [144]. In proliferative melanoma, miR-211 inhibits the expression of both TGFβ receptors, thus suppressing endogenous TGFβ signaling, which results in the weak metastatic ability of these cells. IL-6 and TNF-α secreted by adipocytes block miR-211 expression, leading to the elevated production of TGFβ receptors and, therefore, a phenotypic switch of melanoma from the proliferative to the highly invasive state [144].

Another microRNA, miR-21, is linked to the interplay between adipose tissue and cancer cells. Several reports indicated the role of this microRNA in the regulation of adipose tissue differentiation as well as its contribution to obesity-associated insulin resistance [145]. It is important to note that miR-21-enriched exosomes secreted by adipose-derived stem cells can potentially promote vascularization and even confer chemoresistance of ovarian cancer cells [146,147]. In the case of melanoma, a steady increase in miR-21 level, resulting from endogenous upregulation of its expression or the uptake of exogenous miR-21-carrying exosomes, is related to the progression of melanocyte malignant transformation towards melanoma [19].

Furthermore, cancer-associated adipocytes led to the upregulation of chemokine ligands (CCLs)—CCL19 and CCL21—levels in the lymph nodes, as well as increased chemokine receptor 7 (CCR7) expression in melanoma cells. Human lymph nodes express CCL21, while melanoma cells produce its receptor, CCR7, which is a key molecule mediating metastasis of many cancers. Its overexpression stimulates the metastasis of B16F1 mouse melanoma cells into the lymph nodes [130,148]. Adipocytes also support the bone metastasis of melanoma. Chen et al. demonstrated that a high-fat diet led to an elevated level of bone marrow adipocytes, which was accompanied by IL-6-JAK2-osteopontin-mediated tumor growth, macrophage accumulation, and osteoclastogenesis, predisposing melanoma cells to form metastases in the bone. Blockade of IL-6 or JAK2 inhibited high-fat-diet-stimulated tumor progression [149].

Another type of secreted factors that can support cancer progression is adipocyte-derived exosomes. These vesicles are often enriched in proteins involved in fatty acid oxidation (FAO), a pathway upregulated in advanced melanoma [150]. Exosomes taken up by melanoma cells stimulate metabolic reprogramming enhancing FAO, which promotes cell invasion. In obese individuals, more exosomes filled with fatty acids are secreted by adipocytes, resulting in elevated mitochondrial activity, which contributes to the re-localization of these structures to the membrane protrusions formed by migrating cells. Together, these processes exert a stronger promigratory effect on melanoma cells (Figure 4) [151,152,153].

### 4.4. Epithelial–Mesenchymal Transition

The increased invasive ability of melanoma is also boosted by the elevated expression of the EMT-associated genes triggered by the adipocyte-conditioned medium [154,155]. EMT can be associated with the reduced expression of suppressors such as *KISS1*, which inhibit the invasive abilities of melanoma (Figure 4) [156]. Kushiro et al. showed that higher invasiveness of B16BL6 mouse melanoma cells treated with conditioned medium derived from adipocytes was connected to the increased expression of IL-6 and EMT-associated genes encoding, e.g., Snail, MMP9, Twist, and vimentin (Figure 4). Additionally, in these cancer cells, the expression of E-cadherin and the metastasis suppressor gene *KISS1* were downregulated [157].

Moreover, transformed melanocytes can dedifferentiate through a process similar to the epithelial–mesenchymal transition, which makes their phenotype more aggressive and is linked to a decrease in melanin synthesis by these cells [158,159]. Melanocytes stimulated by adipocyte-derived IL-6 reduce melanogenesis, which supports the thesis concerning the paracrine differentiation of melanoma cells under the influence of adipocytes [121,160].

### 4.5. Adipocytes and Angiogenesis

Adipocytes are also able to support the process of tumor angiogenesis. Coelho et al. showed that CMs derived from adipocytes containing proangiogenic factors like HGF and VEGF were able to induce melanoma vascular mimicry [121]. In addition, in conditioned media collected from both subcutaneous and visceral adipose tissues, endocan—a proinvasive and proangiogenic marker typically overexpressed in tumor vessels—was upregulated (Figure 4) [121,161]. Similar data were previously obtained by Wagner et al. and Jung et al., showing that melanoma angiogenesis can be stimulated by factors supplied by the adipose tissue [130,162]. Brandon et al. also indicated that B16F10 melanoma tumors inoculated into obese mice demonstrated higher VEGF levels and vascularization, while a shift from a high-fat diet to a normal one led to reduced tumor size and rate of vascularization in melanoma-bearing mice [128].

### 4.6. Effect of Adipocytes on Drug Resistance

The presence of adipocytes within the tumor microenvironment can also be associated with the emergence of drug resistance in melanoma cells. Malvi et al. indicated that obesity induced by a high-fat diet reduced the efficacy of dacarbazine (DTIC)-based therapy and decreased the overall survival of melanoma-bearing mice, which was connected to the limited tumor tissue accessibility of DTIC. Moreover, the impaired response of cancer cells to DTIC in an obese mouse model was accompanied by the elevated expression of fatty acid synthase, Cav-1, and P-glycoprotein (P-gp), which is a multidrug resistance protein associated with pumping out drugs from targeted cells. Inhibition of these molecules reversed the chemoresistant phenotype of melanoma (Figure 4) [163].

Additionally, Chi et al. indicated that the incubation of melanoma cells with adipocyte-derived conditioned media reduced the level of apoptosis induced by cisplatin, docetaxel, and the histone deacetylase inhibitor SAHA, which was mediated by a mechanism based on PI3K/AKT and MEK/ERK signaling [164]. Adipocytes can also influence the immune response of the organism to melanoma. It was shown that white adipocytes highly express PD-L1, which can interact with the PD-1 molecule on the surface of T lymphocytes and, in this way, help in cancer cell immune escape. Wu et al. demonstrated that, following the knockout of adipocyte-specific PD-L1 in mice, the immune response directed against the tumor was enhanced [165].

Finally, the upregulation of fatty acid oxidation is one of the requirements for BRAF-mutant melanoma cells to avoid apoptosis under the metabolic stress induced by MAPK inhibitors prior to the emergence of drug resistance [166]. As was mentioned before, FAO can be facilitated by adipocyte-derived exosomes enriched in fatty acids [151]. However, it can also be mediated by the increase in fatty acid receptor expression, CD36, on the melanoma cell surface [166]. Inhibition of this molecule was shown to impair metabolically induced metastasis of melanoma in a mouse model [167].

### 4.7. Influence of Melanoma on Adipocyte Differentiation

The relationship between adipocytes and melanoma cells can work in both directions. Lunavat et al. showed that melanoma cells secrete a distinct subset of extracellular vesicles containing microRNAs associated with cancer progression, including miR-214-3p [68]. This particular non-coding RNA is thought to be involved in adipocyte differentiation through the transcriptomic regulation of the Wnt/β-catenin pathway. It was also demonstrated that overexpression of miR-214-3p in 3T3-L1 mouse preadipocytes led to the upregulation of genes, implicated in lipogenesis, encoding FABP4 (fatty acid-binding protein 4), PPARγ (peroxisome proliferator-activated receptor γ), and adiponectin (Figure 4) [168]. Taken together, these results suggest that melanoma cells could be responsible for adipocyte differentiation, thus facilitating the formation of a more cancer-supportive niche. However, a direct link between melanoma-derived miR-214-3p and preadipocytes has yet to be confirmed.

Another miRNA involved in 3T3-L1 mouse preadipocytes differentiation is let-7, which regulates the shift from clonal expansion of these cells to terminal differentiation [169]. Xiao et al. have shown that melanoma-derived exosomes are enriched in let-7 family miR, namely, let-7i, which is also able to induce the EMT-resembling process in primary melanocytes, thus promoting their invasive abilities [170].

## 5. Conclusions

In the past, cancer cells were the key target during anti-cancer therapy. Today we know that the cells present in the tumor microenvironment also play a significant role in cancer progression. The melanoma tumor microenvironment consists of cellular components including CAFs, keratinocytes, adipocytes, and immune cells, as well as the ECM and physical factors like hypoxia and the pH of the tumor niche. In this review, we have focused on the role of cells present in the tumor in melanoma progression. CAFs and adipocytes support cancer invasion and migration, as well as proliferation through secreted proteins and the production of high-energy metabolites, whereas keratinocytes participate in the regulation of melanomagenesis. These cells influence cancer cells, both through direct interactions and in a paracrine manner. However, it is important to note that all types of described cells are involved in drug resistance acquisition; therefore, they should be considered potential targets in melanoma treatment.

## Data Availability

Data sharing not applicable.
